# Two new species of *Trichocomaceae* (*Eurotiales*), accommodated in *Rasamsonia* and *Talaromyces* section *Bacillispori*, from the Czech Republic

**DOI:** 10.1038/s41598-023-42002-7

**Published:** 2023-09-09

**Authors:** Milan Špetík, Aleš Eichmeier, Jana Burgová, Jos Houbraken

**Affiliations:** 1 Mendeleum–Institute of Genetics, Mendel University in Brno, Valtická 334, 691 44 Lednice na Moravě, Czech Republic; 2 Department of Breeding and Propagation of Horticultural Plants, Mendel University in Brno, Valtická 334, 691 44 Lednice na Moravě, Czech Republic; 3 Westerdijk Fungal Biodiversity Institute, Uppsalalaan 8, 3584 CT Utrecht, The Netherlands

**Keywords:** Fungi, Fungal biology, Fungal evolution, Fungal genetics

## Abstract

During a previous study on microfungi associated with clematis roots, Penicillium-like fungi were isolated and identified based on morphology. In this study, we subjected those strains to a detailed examination which led to the proposal of two taxonomic novelties, named *Rasamsonia chlamydospora* and *Talaromyces clematidis*. The first taxon is characterized by rough-walled mycelium, acerose to flask shaped phialides, cylindrical conidia and by production of chlamydospore-like structures. The four-loci-based phylogeny analysis delineated the taxon as a taxonomic novelty in *Rasamsonia. Talaromyces clematidis* is characterized by restricted growth on Czapek yeast extract agar, dichloran 18% glycerol agar and yeast extract sucrose agar, and production of yellow ascomata on oatmeal agar. Phylogenetic analyses placed this taxon as a taxonomic novelty in *Talaromyces* sect. *Bacillispori*. Both taxa are introduced here with detailed descriptions, photoplates and information on their phylogenetic relationship with related species.

## Introduction

*Trichocomaceae* species represent a diverse group of worldwide distributed fungi occurring in a diverse range of habitats, from soil to vegetation to air, indoor environments, and various food products^1^. Some species are associated with food spoilage^2^ and mycotoxin production (*e.g.*, luteoskyrin, patulin, rubratoxins, viriditoxin)^3,4^; while others are being used or have the potential to be used in biotechnology for enzyme production ^5–8^. The family currently accommodates eight accepted genera: *Acidotalaromyces*, *Ascospirella*, *Dendrosphaera*, *Rasamsonia*, *Sagenomella*, *Talaromyces*, *Thermomyces* and *Trichocoma*^1^.

The genus *Rasamsonia* was established in 2012^9^ and currently contains 14 accepted species^10^: *Rasamsonia aegroticola*, *R. argillacea*,* R. brevistipitata*,* R. byssochlamydoides*,* R. columbiensis*,* R. composticola*,* R. cylindrospora*, *R. eburnea*,* R. emersonii*,* R. frigidotolerans*,* R. oblata*,* R. piperina*,* R. pulvericola* and *R. sabulosa*. One of the hallmarks of *Rasamsonia* was its thermotolerant and thermophilic nature, a character used to distinguish it from the related genus *Talaromyces*. Recently, also the mesophilic species *R. frigidotolerans* and *R. pulvericola* were described in the genus, resulting in the genus’ expanded temperature growth range^11,12^. Nevertheless, the genus is morphologically distinct from phenotypically related genera (e.g., *Paecilomyces*, *Talaromyces*) by the production of olive-brown conidia, cylindrical phialides that usually gradually taper towards the apices and conidiophores with distinctly rough-walled stipes. Four species produce a sexual morph and these ascomata have a scanty covering^1^.

The genus *Talaromyces* was introduced for sexually reproducing *Penicillium* species, that produce soft-walled ascomata covered with interwoven hyphae^1^. Phylogenetic analysis revealed that *Penicillium* subgenus *Biverticillium* and *Talaromyces* together form a monophyletic clade. Currently, *Talaromyces* accommodates sexual and asexual reproducing species and is divided into eight sections, *Bacillispori*, *Helici*, *Islandici*, *Purpurei*, *Subinflati*, *Talaromyces*, *Tenues* and *Trachyspermi*^13,14^. *Talaromyces* sect. *Bacillispori* contains seven accepted species (*Talaromyces bacillisporus*,* T. columbiensis*,* T. emodensis*, *T. hachijoensis*,* T. mimosinus*,* T. proteolyticus* and *T. unicus*) and these species grow restrictedly on Czapek yeast extract agar (CYA), dichloran 18% glycerol agar (DG18), yeast extract sucrose agar (YES), creatine sucrose agar (CREA) and produce mono- to biverticillate conidiophores. With exception of *T. proteolyticus*, all species produce creamish white to yellow ascomata^1^.

In previous studies, microfungi associated with the upper parts (stems, leaves)^15^ and roots^16^ of *Clematis* L. plants were studied. During the analysis of the roots, a novel species named *Paecilomyces clematidis* was described, while several other isolates remained unidentified. In present study, we subjected those isolates to a detailed examination. Two putative new *Trichocomaceae* species were identified, accommodated in *Rasamsonia* and *Talaromyces* sect. *Bacillispori.* We introduce these new species here, and provide detailed descriptions, illustrations, and present data on their phylogenetic relationships with related species.

## Materials and methods

### Collection and isolation

Experimental research and field studies on plants, including the collection of plant material, was complied with relevant institutional, national, and international guidelines and legislation. During the spring of 2021 roots of *Clematis* L. were collected from the ornamental garden (48° 47′ 33.4″ N; 16° 47′ 55.7″ E) of Mendel University in Lednice, the Czech Republic. Collected roots were immediately transported to the laboratory for further processing. The roots were washed with running tap water to remove residual soil, washed again with running sterile distilled water and air-dried on sterile filter paper. Clean roots were sterilized in 1% sodium hypochlorite for one minute and then rinsed three times with sterile distilled water. The disinfected roots were cut into small segments of 2 × 2 × 10 mm and aseptically transferred onto potato dextrose agar (PDA, HiMedia, Mumbai, India) supplemented with 0.5 g L^−1^ streptomycin sulfate (Sigma–Aldrich, St. Louis, MO, USA). The plates were incubated at 25 °C in the dark for six weeks and checked for fungal growth every day. Newly developed colonies were immediately transferred onto new PDA plates and these initial cultures were subsequently purified by single spore isolation^17^. Reference strains and dry specimens are maintained in the CBS culture collection/fungarium and the working collection of the Food and Indoor Mycology research group (DTO), both housed at the Westerdijk Fungal Biodiversity Institute (Utrecht, the Netherlands), and at Fungal Culture Collection of Mendeleum (MEND-F), Mendel University in Brno (Lednice, Czech Republic). Taxonomic novelties were submitted to Mycobank (https://www.mycobank.org).

### Morphology

Culture characteristics were determined after seven days of cultivation in darkness at 25 °C for the *Talaromyces* strains, and at 37 °C for the *Rasamsonia* strains. The strains were inoculated in three equidistant points on CREA, CYA, DG18, malt extract agar (MEA), oatmeal agar (OA) and CYA supplemented with 5% NaCl (CYAS). All media were prepared as previously described^18^. Colony diameters were measured after 7 d (both species) and 14 d (*Talaromyces* species only). The strains were also grown on MEA for 7 d and 14 d at 15, 18, 21, 24, 25, 27, 30, 33, 36, 37, 40, 45, 48, 50, 52 and 55 °C in darkness, to determine cardinal temperatures. Pictures of colonies were captured by Nikon D3200 camera equipped with Nikon 18–55 mm f/3.5–5.6 G AF-P DX VR optics. The picture processing and preparation of photographic plates was done in Adobe Photoshop CS 2018. From 7-day-old cultures grown at 25 °C (*Talaromyces*) and 37 °C (*Rasamsonia*) a conidiogenous layer with conidia were mounted in 60% lactic acid. Excess amounts of conidia were washed out with 70% EtOH. A compound ZEISS AxioSkop 2 microscope equipped with Nikon DS-Ri2 camera was used for bright-field digital images of the micromorphological features. Nikon NIS-Elements D software package was used for capturing pictures and taking measurements.

### DNA extraction and amplification

Genomic DNA was extracted from 7-day-old mycelium grown on MEA at 25 °C (*Talaromyces*) and 37 °C (*Rasamsonia*) in darkness, using a NucleoSpin DNA extraction kit (Macherey–Nagel, Düren, Germany) following the manufacturer’s protocol. The internal transcribed spacer regions incl. 5.8S rDNA (ITS), and parts of the beta-tubulin (*BenA*), calmodulin (*CaM*), second largest subunit of nuclear RNA polymerase II (*rpb2*) and the large ribosomal subunit (LSU) were amplified by PCR using the primers and conditions previous described^16,18^. Sequencing was conducted in both directions with the same primer pair as the primers used for amplification at Eurofins Genomics Germany GmbH (Ebersberg, Germany). Sequences were edited and assembled in Geneious Prime 2022.1.1 (https://www.geneious.com). Newly generated sequences were deposited in GenBank.

### Phylogenetic analysis

Additional sequences were downloaded from GenBank and subjected to phylogenetic analyses together with newly obtained sequences (Table 1). The dataset for each gene was aligned using the MAFFT v. 7 using the European Bioinformatics Institute platform (EMBL-EBI, https://www.ebi.ac.uk)^19^. Obtained alignments were manually checked, edited and combined using MEGA v.7^20^. The combined ITS, *BenA*,* CaM* and *rpb2* dataset was subjected to Maximum likelihood (ML) analyses. Phylogenetic trees were constructed using IQ-TREE 2^21^, running 1000 bootstrap replicates. The best models for ML analyses were selected based on the Bayesian information criterion (BIC) calculated in IQ-TREE 2. Bayesian analyses (BI) employed MrBayes v. 3.2.7^22,23^. The BI analyses included four parallel runs of 50 M generation starting from a random tree topology, every 1000 generations were sampled and the first 25% of the trees were discarded as the ‘burn-in’. The most suitable substitution model for BI analysis was determined separately for each loci using jModelTest v. 2.1.7^24^. Trees were visualized in FigTree v. 1.4.4 and edited in Adobe Illustrator CC 2019. Resulted trees of both methods shared similar topology, thus we decided to present ML trees with support values of both methods –bootstrap (BS) and posterior probabilities (pp) labelled at the nodes. Values below 0.95 (pp) and 75% (BS) support are not shown or indicated with a hyphen. The alignments and corresponding trees are available at Figshare (10.6084/m9.figshare.20490051).

**Table 1:** Fungal species and barcodes used in the phylogenetic analyses.

Species	Strain no	ITS	*BenA*	*CaM*	*rpb2*
*Rasamsonia aegroticola*	CBS 132819^ T^ = DTO 137-A8 = IHEM 22641	JX272988	JX273020	JX272956	MN969193
*R. argillacea*	CBS 101.69^ T^ = IHEM 22033	JF417491	JF417456	JF417501	JF417415
*R. brevistipitata*	CBS 128785^ T^ = DTO 025-H2 = IBT 31187	JF417488	JF417454	JF417499	JN406530
*R. byssochlamydoides*	CBS 413.71^ T^ = DTO 149-D6 = IBT 11604	JF417476	JF417460	JF417512	JF417437
*R. chlamydospora*	CBS 149229^ T^ = DTO 473-E5 = MEND-F-0752	**ON863770**	**ON873765**	**ON938198**	**ON938202**
*R. chlamydospora*	CBS 148468 = DTO 473-E4 = MEND-F-0751	**ON863769**	**ON873764**	**ON938197**	**ON938201**
*R. columbiensis*	CCF 5289^ T^	LT548281	LT548285	MN969326	MN969195
*R. composticola*	CGMCC 3.13669^ T^	JF970184	JF970183	JQ729688	JQ729684
*R. cylindrospora*	CBS 275.58^ T^ DTO 138-F8 = IBT 31202	JF417470	JF417448	JF417493	JF417423
*R. eburnea*	CBS 100538^ T^ = DTO 105-D6 = IBT 17519	JF417483	JF417462	JF417494	JN406532
*R. emersonii*	CBS 393.64^ T^ = ATCC 16479 = IMI 116815 = IMI 116815ii = IBT 31218 = IBT 21695	JF417478	JF417463	JF417510	XM013471581
*R. frigidotolerans*	FMR 16675^ T^	LT985886	LT985895	LT985897	–
*R. oblata*	NBRC 33091^ T^	LC546728	LC546717	LC546739	–
*R. piperina*	CBS 408.73^ T^ = DTO 138-G3 = IJFM 1326	JX272968	JX273000	JX272936	MN969194
*R. pulvericola*	DAOM 242435^ T^	KF242514	KF242520	KF242522	KF242518
*R. sabulosa*	ATCC 56984^ T^	LC546720	LC546726	LC546742	–
*Talaromyces bacillisporus*	CBS 296.48^ T^ = IMI 040045 = NRRL 1025	KM066182	AY753368	KJ885262	JF417425
*T. clematidis*	CBS 149228^ T^ = DTO 473-E3 = MEND-F-0750	**ON863768**	**ON873763**	**ON938196**	**ON938200**
*T. clematidis*	CBS 148467 = DTO 473-E2 = MEND-F-0749	**ON863767**	**ON873762**	**ON938195**	**ON938199**
*T. columbiensis*	CBS 113151^ T^ = IBT 23206 = DTO 058-F3	KX011503	KX011488	KX011499	MN969187
*T. emodensis*	CBS 100536^ T^ = IBT 14990	JN899337	KJ865724	KJ885269	JF417445
*T. flavus*	NRRL 2098^ T^ = IMI 197477 = NRRL 2098	JN899360	JX494302	KF741949	JF417426
*T. hachijoensis*	CBM-FA-0948^ T^	AB176620	–	–	–
*T. mimosinus*	CBS 659.80^ T^ = FRR 1875 = IMI 223991	JN899338	KJ865726	KJ885272	MN969149
*T. proteolyticus*	CBS 303.67^ T^ = NRRL 3378	JN899387	KJ865729	KJ885276	KM023301
*T. subinflatus*	CBS 652.95^ T^ = IBT 17520	JN899397	MK450890	KJ885280	KM023308
*T. unicus*	CBS 100535^ T^ = IBT 18385 = FRR 4436	JN899336	KJ865735	KJ885283	MN969150
*Trichocoma paradoxa*	CBS 103.73^ T^ = IBT 31160	MH860643	JF417469	JF417506	JN121417

Newly generated sequences are highlighted in bold.

*ATCC* American Type Culture Collection (Virginia, USA); *CBM* Natural History Museum and Institute, Chiba (Japan); *CBS* CBS culture collection housed at Westerdijk Fungal Biodiversity Institute (WI) (the Netherlands); *CCF* Culture Collection of Fungi (Czech Republic); *CGMCC* China General Microbiological Culture Collection (P.R. China); *DTO* Working collection of Food and Indoor Mycology department housed at WI; *DAOM* Canadian National Mycological Herbarium (Ontario, Canada);T *FMR* Facultat de Medicina, Universitat Rovira i Virgili, Reus (Spain); *IBT* Culture collection of Center for Microbial Biotechnology (CMB) at the Department of Systems Biology, Technical University of Denmark; *IHEM* Culture collection of the Scientific Institute of Public Health Mycology Section (Brussels, Belgium); *IJFM* Instituto ´Jaime Ferrán´ de Microbiología Consejo Superior de Investigaciones Científicas, (Madrid, Spain); *MEND-F* Fungal Culture Collection of Mendeleum, Mendel University in Brno (Czech Republic); *NBRC* Biological Resource Center, NITE (Japan); *NRRL* ARS Culture Collection (Illinois, USA).

^T^ex-type strain.

## Results

### Phylogenetic analysis

The ML analyses based on the *rpb2* dataset (Fig. S1) showed the phylogenetic placement of strains CBS 149229, CBS 148468, CBS 148467 and CBS 149228 within *Trichocomaceae*. Detailed statistics and the model selected for the ML analysis is given in Table 2. The phylogram was rooted with *Paecilomyces brunneolus* CBS 370.70^ T^. Briefly, the ML analysis grouped studied strains into 15 lineages, including outgroup. These lineages are in agreement with data published in studies on *Trichocomaceae*^13,14,25^. Based on these data, CBS 149229 and CBS 148468 can be classified in *Rasamsonia*, and CBS 148468 and CBS 148467 in *Talaromyces* sect. *Bacillispori*.

**Table 2:** Detailed characteristics of phylogeny datasets.

Dataset	Locus	No. of sequences	Sites	Parsimony-informative	Constant sites	ML model	BI model
*Rasamsonia*	ITS	17	615	108	434	TN + F + G4	SYM + G
*Rasamsonia*	*BenA*	17	490	149	263	K2P + I	SYM + G
*Rasamsonia*	*CaM*	17	559	178	310	K2P + I	HKY + I + G
*Rasamsonia*	*rpb2*	14	960	206	664	TIM2e + G4	SYM + G
*Talaromyces*	ITS	11	550	87	392	TN + F + G4	HKY + I + G
*Talaromyces*	*BenA*	10	459	120	278	TPM2 + G4	SYM + G
*Talaromyces*	*CaM*	10	527	153	283	K2P + G4	SYM + G
*Talaromyces*	*rpb2*	10	976	181	679	TNe + G4	SYM + I + G
*Trichocomaceae*	*rpb2*	141	1006	441	541	TIM3e + R7	–

**Figure 1:**
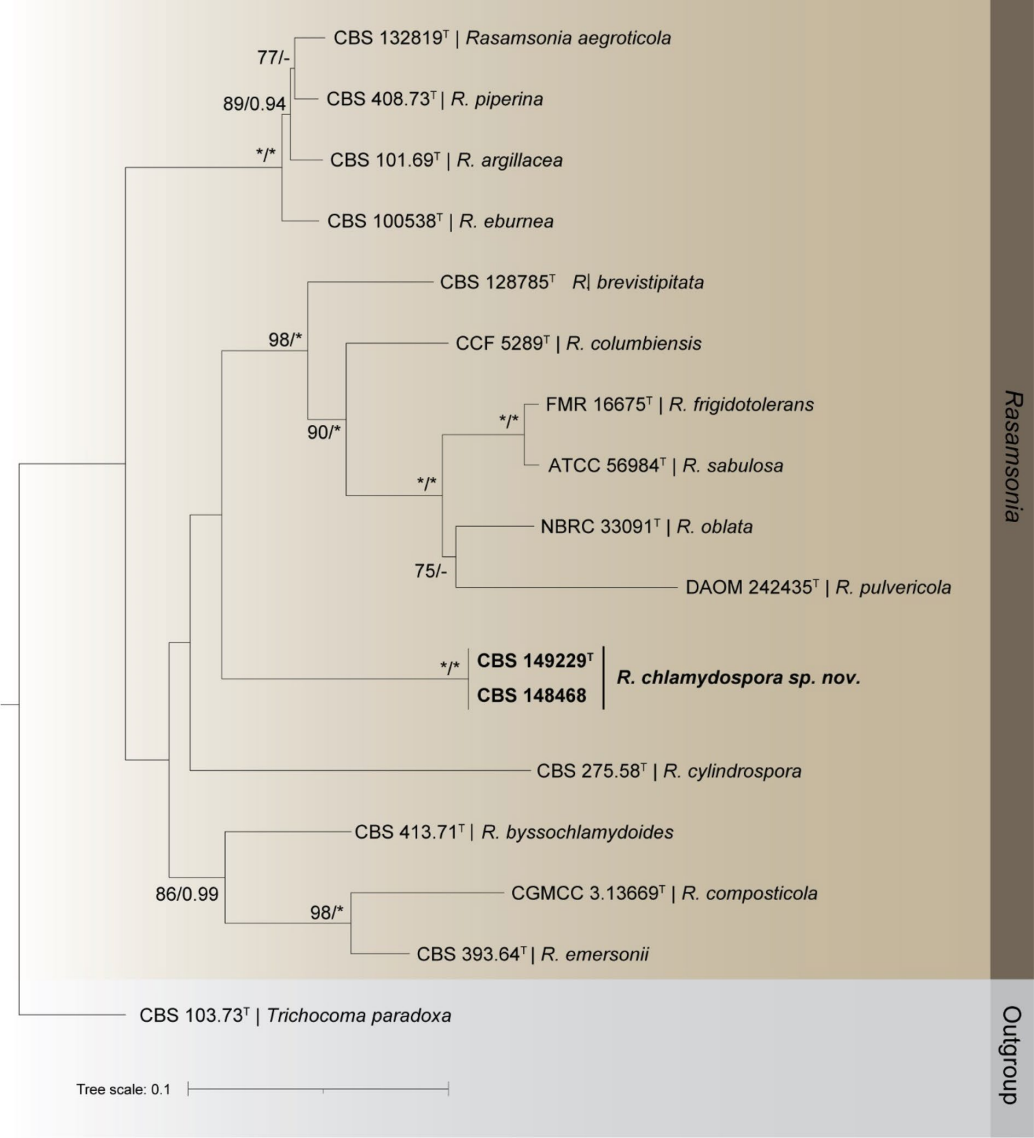
Maximum likelihood tree generated from the combined analysis of ITS, *BenA*, and *CaM* sequence data. BS/pp values are given at the nodes. The tree was rooted to *Trichocoma paradoxa* (CBS 103.73^ T^). The new species *Rasamsonia chlamydospora* Spetik & Houbraken *sp. nov.* is highlighted in bold. ^T^ex-type strain.

The *Rasamsonia* dataset contained sequences from 17 strains, including the outgroup *Trichocoma paradoxa* (CBS 103.73^ T^). The combined dataset (ITS, *BenA*, and *CaM*) contained 1664 sites, including alignment gaps. Of these, 1007 were conserved and 435 parsimony-informative. Detailed results for each single gene dataset including corresponding models are given in Table 2. Strains CBS 148468 and CBS 149229 form a lineage within *Rasamsonia* (Fig. 1). This lineage is sister to a clade containing *R. brevistipitata*, *R. columbiensis*, *R. frigidotolerans*, *R. oblata*, *R. pulvericola* and *R. sabulosa*. Single locus trees of individual loci (ITS, *BenA*, *CaM*, *rpb2*) are shown in Fig. S2.

**Figure 2:**
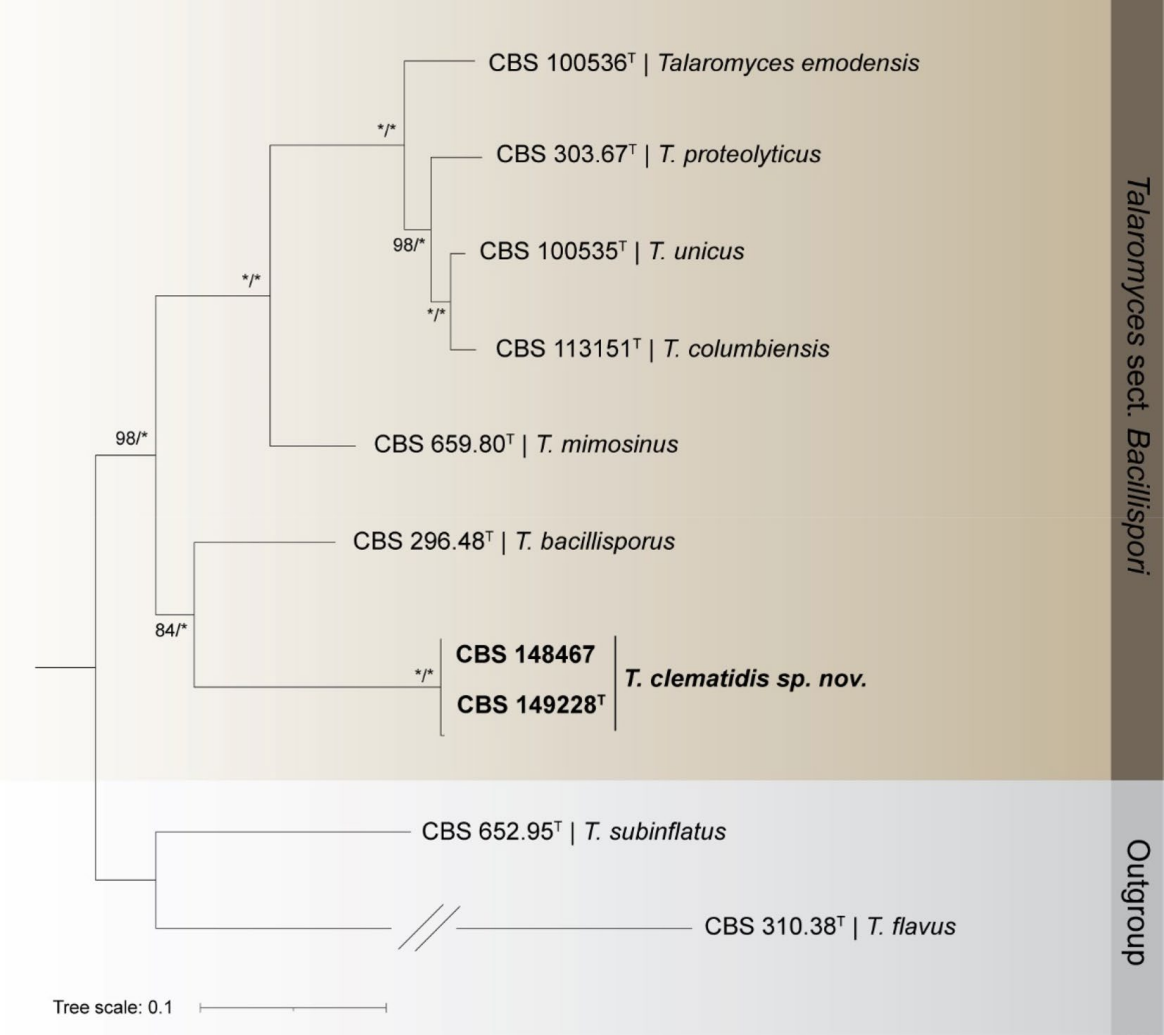
Maximum likelihood tree generated from the combined analysis of ITS, *BenA*, *CaM* and *rpb2* sequence data. BS/pp values are given at the nodes. The tree was rooted to *Talaromyces subinflatus* (CBS 652.95^ T^) and *Talaromyces flavus* (CBS 310.38^ T^). The new species *Talaromyces clematidis* Spetik & Houbraken *sp. nov.* is highlighted in bold. ^T^ex-type strain.

The *Talaromyces* sect. *Bacillispori* dataset consisted of sequences from 10 strains, including the outgroups *Talaromyces subinflatus* (CBS 652.95^ T^) and *Talaromyces flavus* (CBS 310.38^ T^). The combined dataset (ITS, *BenA*, *CaM* and *rpb2)* contained 2512 sites, including alignment gaps. Of these, 1632 were conserved, 541 parsimony-informative and 817 unique. Detailed results for each single gene dataset including corresponding models are displayed in Table 2. The multilocus analysis resolved CBS 148467 and CBS 149228 in a fully supported clade, sister to *T. bacillisporus* CBS 296.48^ T^ (84% BS, 1.00 pp) (Fig. 2). Additionally, a single locus ITS ML/BI analysis was performed (Fig. S3), showing the distant position of *T. hachijoensis* to the newly proposed *T. clematidis.*

### Taxonomy

Based on the results of the phylogenetic analysis and the morphological examination (see below under Taxonomy and Discussion), we propose the names *Rasamsonia chlamydospora* for CBS 148468 and CBS 149229 and *Talaromyces clematidis* for CBS 148467 and CBS 149228.

**Figure 3:**
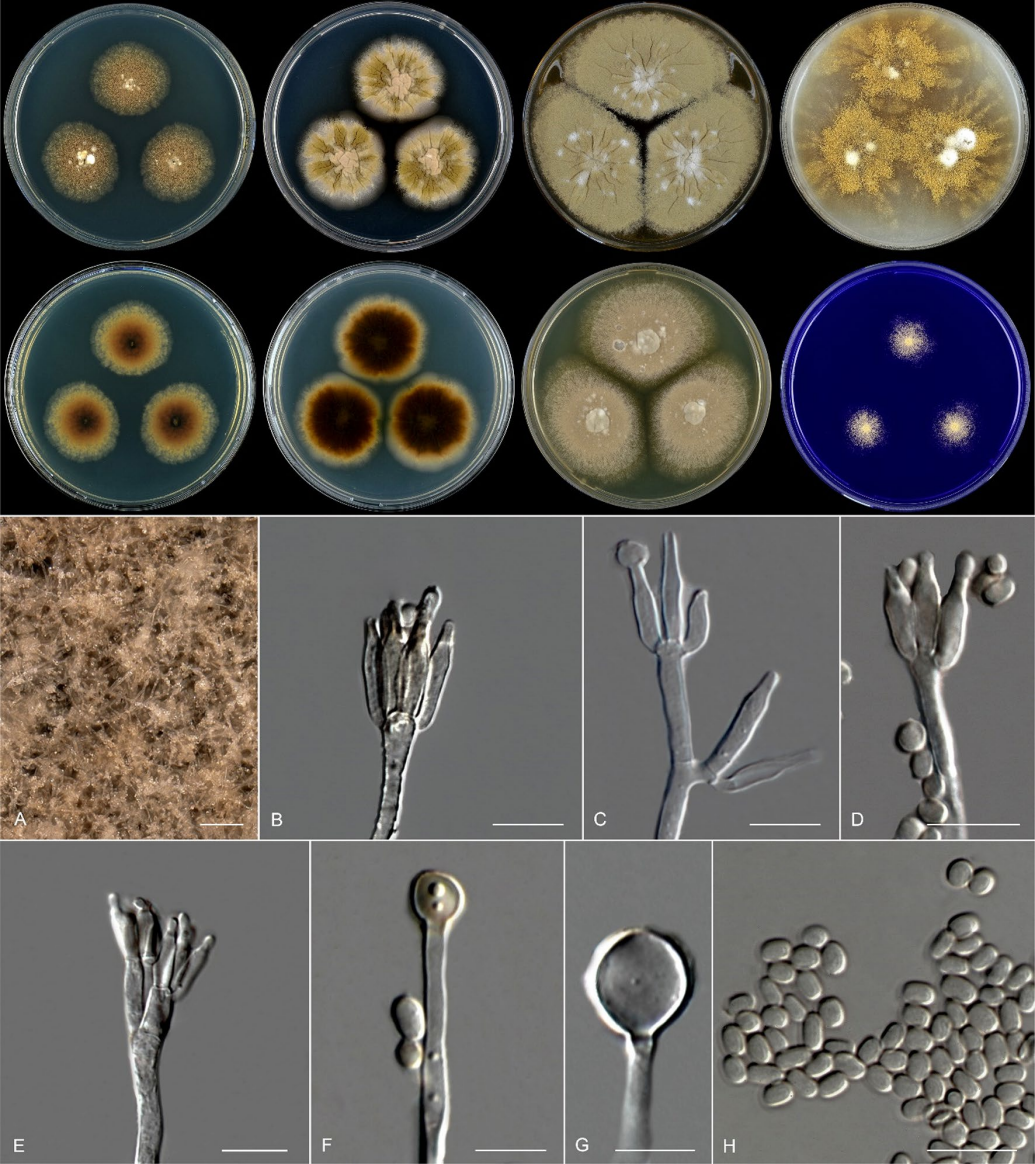
*Rasamsonia chlamydospora* (CBS 149229^ T^). Colonies 7 d, 37 °C from left to right (top row) CYA, DG18, MEA and OA; (bottom row) CYA reverse, DG18 reverse, YES and CREA. (**A**) In situ detail of the colony on MEA; (**B**–**E**) Conidiophores; (**F**, **G**) Chlamydospores; (**H**) Conidia. Scale bars: 500 μm (**A**); 10 μm (**B**–**H**); 5 μm (**G**).

***Rasamsonia chlamydospora*** Spetik & Houbraken *sp. nov.* (Fig. 3).

Mycobank number: MB 845365.

**Etymology:**—refers to chlamydospores, the globose to subglobose swollen cells produced by this species.

Type:—**CZECH REPUBLIC**, Břeclav: Lednice, university garden (48° 47′ 33.4″ N 16° 47′ 55.7″ E), isolated from the root of *Clematis* ´Snow Queen´ (*Ranunculaceae*), 2021, M. Spetik, Holotype: CBS H-25023, ex-type living culture CBS 149229 = DTO 473-E5 = MEND-F-0752.

**Barcodes:** ITS ON863770; LSU ON863795; *BenA* ON873765; *CaM* ON938198; *rpb2* ON938202.

*Colony diameter (7 d*,* in mm)*: 25 °C: CYA 10–13; CYAS No growth; DG18 2; MEA 25–28; OA 12–21; YES 10–12; CREA No growth. 37 °C: CYA 31–35; CYAS No growth; DG18 36–38; MEA > 65; OA > 65; YES 46–52; CREA 18–25.

*Colony diameter at different temperatures (7 d in mm). On CYA*: 15–21 °C No growth; 24 °C 5–7; 27 °C 16–18; 30 °C 20–23; 33 °C 27–30; 36 °C 28–33; 37 °C 31–35; 40 °C 26–29; 45 °C 15–16; 48 °C 7–8; > 50 °C No growth. *On MEA*: 15–18 °C No growth; 21 °C 9–11; 24 °C 25–27; 27 °C 45–46; 30 °C 56–58; 33 °C 66–71; 36 °C 72–76; 37 °C 70–74; 40 °C 75; 45 °C 39–45; 48 °C 21–25; 50 °C 12–13; 52 °C 5; 55 °C No growth. *Cardinal growth temperatures*: Minimum, on CYA between 21 and 24 °C and between 18 and 21 °C on MEA, optimum around 36 °C and maximum on CYA 48 °C, on MEA 52 °C. Colonies grown on CYA and MEA at various temperatures (15–45 °C) after 7 d are shown on Fig. S4.

*Colony characters (37 °C*,* 7 d)*: CYA: Colonies low, plane; margin low, entire; mycelium light brown; texture floccose; sporulation moderately dense; soluble pigments absent; exudates absent; reverse white to light brown. MEA: Colonies moderately deep, radially sulcate; margin low, entire; mycelium olivaceous with white spots near the centre; texture velvety; sporulation dense; soluble pigments absent; exudates absent; reverse brown. DG18: Colonies raised, sulcate; margin low, entire; mycelium white to olivaceous; texture velvety to floccose; sporulation dense; soluble pigments absent; exudates absent; reverse brown. YES: Colonies flat, raised in centre; margin low, entire; mycelium olivaceous with white spots; texture velvety to floccose; sporulation dense; soluble pigments absent; exudates absent; reverse white. OA: Colonies flat; margin irregular; mycelium yellow to olivaceous; texture floccose; sporulation dense; soluble pigments absent; exudates absent; reverse brown. CYAS: No growth. CREA: Moderate growth, acid production absent.

*Micromorphology*:* Mycelium* 2–3.5 μm diam, distinctly rough-walled. *Conidiophores* monoverticillate, sometimes with subterminal branches. *Stipes* rough-walled, 15–70 × 2–3 μm. *Phialides* 3–6 per stipe, smooth, acerose to flask-shaped, 10–15 × 2–3 μm. *Conidia* smooth, cylindrical, 3.5–4.5 × 2.5–3 μm. Swollen cells resembling chlamydospores present, smooth, globose to subglobose, 6–8 μm. *Ascomata* not observed.

*Notes*: Most *Rasamsonia* species are thermotolerant or thermophilic, including *R. chlamydospora.* Thermophilic fungi are those with a maximum growth temperature of 50 °C or higher, and a minimum growth temperature of 20 °C or above^26^. The minimum growth temperature of *R. chlamydospora* depends on the growth medium, and the minimum growth temperature on MEA is between 18 and 21 °C. Comparison of microscopic features between all *Rasamsonia* species is given in Table 3. *Rasamsonia chlamydospora* shares the production of olive-brown conidia and cylindrical phialides that gradually taper towards the apices with other species but can be distinguished from other *Rasamsonia* species by the production of swollen cells that resemble chlamydospores. Comparing sequences obtained from the type strain with those of *R. brevistipitata*, the phylogenetically closest species, showed a pairwise nucleotide difference of 27 bp in ITS, 63 bp in *BenA*, 79 bp in *CaM* and 88 bp in *rpb2.*

**Table 3:** Morphological characteristics of *Rasamsonia* spp.

Species	Colonies 7d [mm]			
	CYA 25 °C	CYA 37 °C	Shape and size of conidia [µm]	Ascomata
*R. aegroticola*	15–25	30–40	Cylindrical or ovoid; (3–)3.5–4.5(–5) × 1.5–2(–2.5)	Absent
*R. argillacea*	15–25	30–40	Cylindrical or ovoid; 3.5–4.5 × 1.5–2	Absent
*R. brevistipitata*	7–13	11–17	Ellipsoidal or ovoid; (2–)2.5–3(–3.5) × 1.5–2	Absent
*R. byssochlamydoides*	No growth	19–27	Cylindrical; 4–8 × 1–2.5	Present
R. chlamydospora	10–13	31–35	Cylindrical; 3.5–4.5 × 2.5–3	Absent
*R. columbiensis*	14–15	15–16	Cylindrical to ovoid; (2.5–)3–4(–7) × 2.5–4.5	Absent
*R. composticola*	n/a	25–40	Cylindrical; 3–9 × 1.5–4	Present
*R. cylindrospora*	3–8	5–10	Cylindrical; 4–5 × 1.5–2	Absent
*R. eburnea*	14–20	30–40	Cylindrical at first, becoming ellipsoidal or ovoid; 2.5–3.5(–4) × 1.8–2.5	Occasionally present
*R. emersonii*	No growth	18–30	Cylindrical; 3.5–4.5(–5.0) × 1.5–3	Present
*R. frigidotolerans*	3–4	No growth	Globose; 1–2	Absent
*R. oblata*	2	3	Globose to subglobose; 2–2.5 × 2.5–3	Absent
*R. piperina*	n/a	15–25	Ellipsoidal or cyclindrical; 2–3.5 × 1.7–2.5	Absent
*R. pulvericola*	3–4	No growth	Subglobose; 2–2.5 × 2–2.5	Absent
*R. sabulosa*	1–2	2	Subglobose; 2–2.5	Absent

Data obtained from references ^9,11,27,28^; n/a: no data available.

**Additional specimens examined:—CZECH REPUBLIC**, Breclav: Lednice, university garden (48° 47′ 33.3″ N 16° 47′ 55.6″ E), isolated from the root of *Clematis* ´Snow Queen´ (*Ranunculaceae*), 2021, M. Spetik, living culture CBS 148468 = DTO 743-E4 = MEND-F-0751.

**Figure 4:**
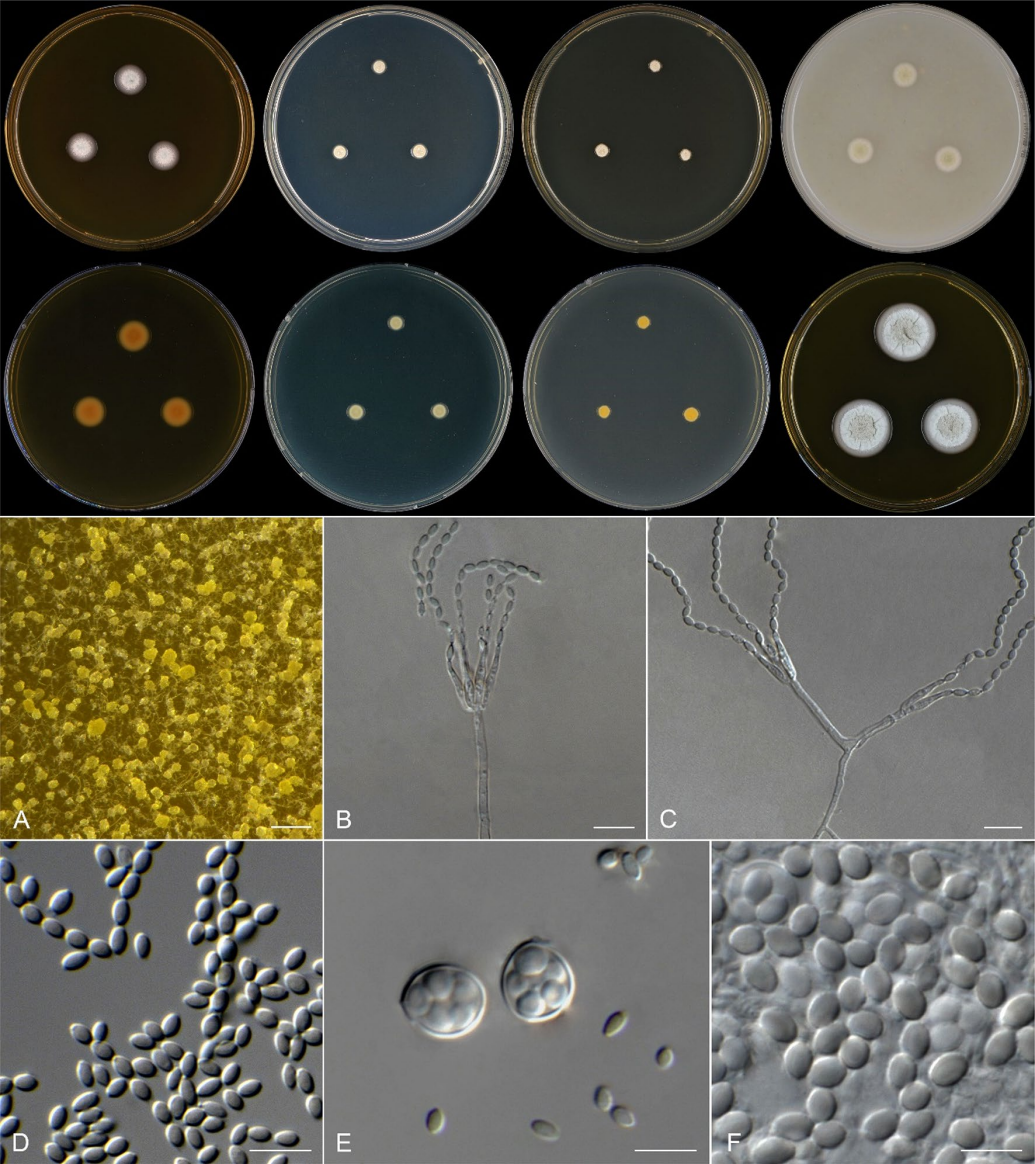
*Talaromyces clematidis* (CBS 149229^ T)^. Colonies 7 d, 25 °C from left to right (top row), MEA, DG18, YES and OA; (bottom row) MEA reverse, DG18 reverse, YES reverse; MEA (14 d 25 °C). (**A**) Ascomata in situ on OA; (**B**, **C**) Conidiophores; (**D**) Conidia; (**E**) Asci and conidia; (**F**) Ascospores. Scale bars: 1000 μm (**A**), 10 μm (all others).

***Talaromyces clematidis*** Spetik & Houbraken *sp. nov*. (Fig. 4).

Mycobank number: MB 845200.

**Etymology:—**refers to the host, *Clematis*.

Type:—**CZECH REPUBLIC**, Břeclav: Lednice, university garden (48°47′ 33.5″ N 16° 47′ 55.8″ E), isolated from the root of *Clematis* ´Snow Queen´ (*Ranunculaceae*), 2021, M. Spetik, Holotype: CBS H-25024, ex-type living culture CBS 149228 = DTO 473-E3 = MEND-F-0750.

**Barcodes:** ITS ON863768; LSU ON863793; *BenA* ON873763; *CaM* ON938196; *rpb2* ON938200.

*Colony diameter (25 °C*,* in mm)*, *7 d*: CYA 2; CYAS No growth; DG18 3–5; MEA 8–11; OA 6–9; YES 4–6; CREA 25 °C No growth. *14 d*: CYA 4–5; CYAS No growth; DG18 10–13; MEA 21–26; OA 15–18; YES 8–9; CREA No growth. Colonies grown on CYA, MEA, DG18, YES, OA at 25 °C after 7 d and 14 d are displayed in (Fig. S5).

*Colony diameter at different temperatures (mm). On CYA*,* 7 d*: 21 °C No growth; 24 °C 2; 27 °C 3–4; 2; 33 °C 2; 36 °C No growth. *On MEA*,* 7 d*: 15 °C 3–4; 18 °C 3–6; 21 °C 7; 24 °C 3–4; 27 °C 10–12; 30 °C 11–12; 33 °C 8; 36 °C 2; 37 °C No growth. *On CYA 14 d*: 15 °C 2; 18 °C 3–4; 21 °C 3; 24 °C 4; 27 °C 5; 30 °C 4–5; 33 °C 4–5; CYA 36 °C No growth. *On MEA*,* 14 d*: 15 °C 8; 18 °C 12–14; 21 °C 18–21; 24 °C 22–25; 27 °C 25–30; 30 °C 26–28; 33 °C 18–20; 36 °C 7; 37 °C No growth. *Cardinal growth temperatures*: Minimum 15 °C; optimum between 27 and 30 °C and maximum at 36 °C. Colonies grown on CYA and MEA at various temperatures (15–36 °C) after 14 d are displayed on (Fig. S6).

*Colony characters (25 °C*,* 7 d)*: CYA: Growth restricted; colonies flat; margin plane, irregular; mycelium white; texture floccose; sporulation absent; soluble pigments absent; exudates absent; reverse white. MEA: Colonies raised in centre; margin low, entire (2 mm); mycelium white; texture floccose; sporulation dense; soluble pigments absent; exudates absent; reverse white. DG18: Colonies low; margin entire; mycelium white; sporulation absent; soluble pigments absent; exudates absent; reverse pale yellow. YES: Growth restricted; colonies medium raised; margin entire, circular; mycelium white; texture floccose; sporulation absent; soluble pigments absent; exudates absent; reverse white. OA: Colonies flat; margin entire; mycelium white at margin, olivaceous in centre; texture floccose; sporulation dense; soluble pigments absent; exudates absent; reverse white. CYAS: No growth. CREA: No growth.

*Micromorphology*:* Conidiophores* monoverticillate, sometimes with subtermal branches*. Stipes* smooth-walled, 15–30 × 2 μm. *Phialides* usually in groups of 3–6 per stipe, sometimes solitary, lateral of terminal on vegetative hyphae, smooth, acerose, 7–15 × 2–2.5 μm. *Conidia* smooth, ellipsoidal 3.5–5 × 2–3 μm*. Ascomata* yellow, subglobose to ovoidal, 100–600 μm; asci globose to subglobose 11–14 μm; ascospores smooth, ellipsoid, 6–7.5 × 4.5–5.5 μm.

*Notes*:* Talaromyces clematidis* forms a well-supported sister clade to *T. bacillisporus*. There are various characters (e.g., colony diameters on CYA at 25 °C and 37 °C, and ascospore ornamentation) that can be used to distinguish both species, and some of them are summarized in Table4. The pairwise nucleotide differences between the type strains of both species are 45 nucleotides in ITS, 73 bp in *BenA*, 94 bp in *CaM* and 112 bp in *rpb2.*

**Additional specimens examined:**—**CZECH REPUBLIC**, Břeclav: Lednice, university garden (48° 47′ 33.4″ N 16° 47′ 55.7″ E), isolated from the root of *Clematis* ´Snow Queen´ (*Ranunculaceae*), 2021, M. Spetik, living culture CBS 148467 = DTO 743-E2 = MEND-F-0749.

## Discussion

*Rasamsonia chlamydospora* is characterized by production of swollen cells that resemble chlamydospores, which is a unique microscopic feature within *Rasamsonia*. Only *R. byssochlamydoides* was recorded rarely producing chlamydospores that are globose or subglobose, 4 µm diam^29^. In comparison, *R. chlamydospora* produces bigger chlamydospores (6–8 μm), grows faster on CYA and has smaller conidia (Table 3). The four-loci-based phylogeny delineated *R. chlamydospora* as a well-supported clade in *Rasamsonia* (Fig. 1; Fig. S2). *Rasamsonia* species have been reported from various countries, *e.g.* Belgium, Canada, Germany, Italy, Japan, Netherlands, UK, USA, Taiwan; and from various substrates including air, compost, cork, fruit concentrate, human tissues, house dust, peat, seeds, soil, sugar cane, urine or wood chips^9,11,28^. This is for the first-time reporting *Rasamsonia* species from a clematis plant.

*Talaromyces clematidis* is characterized by restricted growth on CYA, DG18 and YES and by production of yellow ascomata. These characters are shared with other *Talaromyces* species accommodated in sect. *Bacillispori* (Table 4)*.* Despite the shared morphology with other *Talaromyces* species, *T. clematidis* forms a well-supported sister clade with *T. bacillisporus* (Fig. 2) employing a four gene (ITS, *BenA*, *CaM*, *rpb2*) dataset. Both species can be distinguished by different conidial shapes, being ellipsoidal in *T. clematidis vs* rod-shaped/ellipsoidal in *T. bacillisporus*. Both species produce ascomata which differ in color; yellow in *T. clematidis*, and white to orange in *T. bacillisporus*. *Talaromyces clematidis* doesn't grow at 37 °C on MEA and CYA while *T. bacillisporus* does. Species of *Talaromyces* sect. *Bacillispori* have been isolated from various substrates such as leaf, rye bread, sludge of anaerobic pasteurised organic household waste, soil, and from several countries including Colombia, Japan, Nepal, Netherlands, Sweden, Ukraine, UK, USA and Taiwan^13,30^. Only one *Talaromyces* strain was isolated from clematis before, in Boskoop, the Netherlands, and this strain (*T. muroi*, CBS 261.55) is accommodated in *Talaromyces* sect. *Talaromyces*^13^. This is the first time that a species of *Talaromyces* sect. *Bacillispori* is reported from clematis.

**Table 4:** Morphological comparison of *Talaromyces* sect. *Bacillispori* species.

Species	Ascomata	Conidial shape	Conidia [μm]	Colonies 7d, [mm]		
				CYA 25 °C	MEA 25 °C	CYA 37 °C
*T. bacillisporus*	Present, creamish white to pastel orange	Cylindrical, rod shaped to ellipsoidal	3–5(–6.5) × 1–2	10–15	15–20	33–37
T. clematidis	Present, yellow	Ellipsoidal	2.5–3.5 × 1.5–2.5	2	8–11	No growth
*T. columbiensis*	Absent	Ovoidal to ellipsoidal	2.5–3 × (1.8–) 2–2.5	15–17	15–17	No growth
*T. emodensis*	Present, creamish white to sulphur yellow	Ovoidal to ellipsoidal	3–4 × 1.5–3	8–10	8–10	4–6
*T. hachijoensis*	Present, yellow	Absent	Absent	3	8–10	No growth
*T. mimosinus*	Present, yellow to sulphur yellow	Globose to subglobose	2–3 × 2–2.5	12–15	13–14	3–5
*T. proteolyticus*	Absent	Globose to subglobose	2–3 × 1.5–2.5	20–22	20–21	No growth
*T. unicus*	Present, yellow	Ellipsoidal to ovoidal	2.7–5 × 1.7–3.2	10.5–16	15–19.5	No growth

References ^13,30^.

## Conclusion

In this study, two novel *Trichocomaceae* species were introduced with comprehensive descriptions, illustrations and taxonomic placement using multi-locus (ITS, *BenA*, *CaM*, *rpb2*) sequence datasets. The proposed novelties are *Rasamsonia chlamydospora* and *Talaromyces clematidis*, accommodated in *Talaromyces* sect. *Bacillispori*, both isolated from a clematis root. Together with our previous study^16^, this study suggests potentially hidden fungal diversity within the root of clematis plants.

### Supplementary Information

Supplementary Legends.

Supplementary Figure S1.

Supplementary Figure S2.

Supplementary Figure S3.

Supplementary Figure S4.

Supplementary Figure S5.

Supplementary Figure S6.

## Data Availability

Newly generated sequences were deposited in NCBI GenBank database under the accession numbers shown in Table 1. The alignments and corresponding trees are available at Figshare (10.6084/m9.figshare.20490051).
