# Supplementary material for: Two new species of Trichocomaceae (Eurotiales), accommodated in Rasamsonia and Talaromyces section Bacillispori, from the Czech Republic
Source: Sci Rep. 2023 Sep 9;13:14903. doi: 10.1038/s41598-023-42002-7 (PMC10492856; doi:10.1038/s41598-023-42002-7)
Supplement: Supplementary file 1 — Supplementary Legends. [file 41598_2023_42002_MOESM1_ESM.pdf]

Figure S1. ML tree of *Trichocomaceae* based on *rpb2* dataset with BP values at the nodes, BP< 75 % are not shown.

Figure S2. Maximum likelihood trees were generated from the analysis of individual single-locus *Rasamsonia* sequence data (ITS, *BenA*, *CaM*, *rpb2*). BS/pp values are given at the nodes. The trees were rooted to Trichocoma paradoxa (CBS 103.73^{T}). The new species *Rasamsonia chlamydospora* Spetik & Houbraken *sp. nov.* is highlighted in bold. ^{T}ex-type strain.

Figure S3. Maximum likelihood tree generated from the analysis of ITS *Talaromyces* sequence data. BS/pp values are given at the nodes. The tree was rooted to *Talaromyces subinflatus* (CBS 652.95^{T}) and *Talaromyces flavus* (CBS 310.38^{T}). The new species *Talaromyces clematidis* Spetik & Houbraken *sp. nov.* is highlighted in bold. ^{T}ex-type strain.

Figure S4. Colonies of *Rasamsonia chlamydospora* on MEA and CYA 7 d at various temperatures (18–52 °C).

Figure S5. Colonies of *Talaromyces clematidis* on various media (DG18, CYA, YES, MEA, OA) at 25 °C after 7 and 14 d.

Figure S6. Colonies of *Talaromyces clematidis* on MEA and CYA after 14 d at various temperatures (15–36 °C).
