# Supplementary figures and images for: Two new species of Trichocomaceae (Eurotiales), accommodated in Rasamsonia and Talaromyces section Bacillispori, from the Czech Republic
Source: Sci Rep. 2023 Sep 9;13:14903. doi: 10.1038/s41598-023-42002-7 (PMC10492856; doi:10.1038/s41598-023-42002-7)

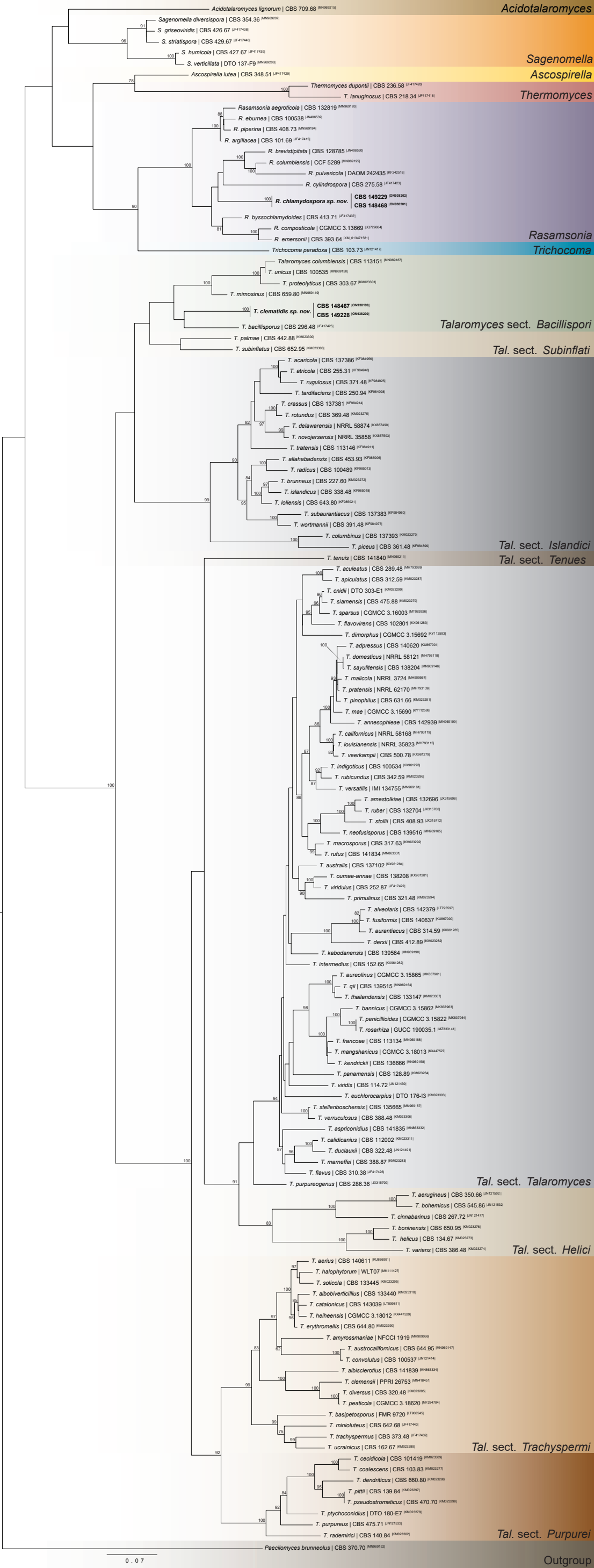

Supplement: Supplementary file 2 — Supplementary Figure S1. [file 41598_2023_42002_MOESM2_ESM.pdf]

ITS

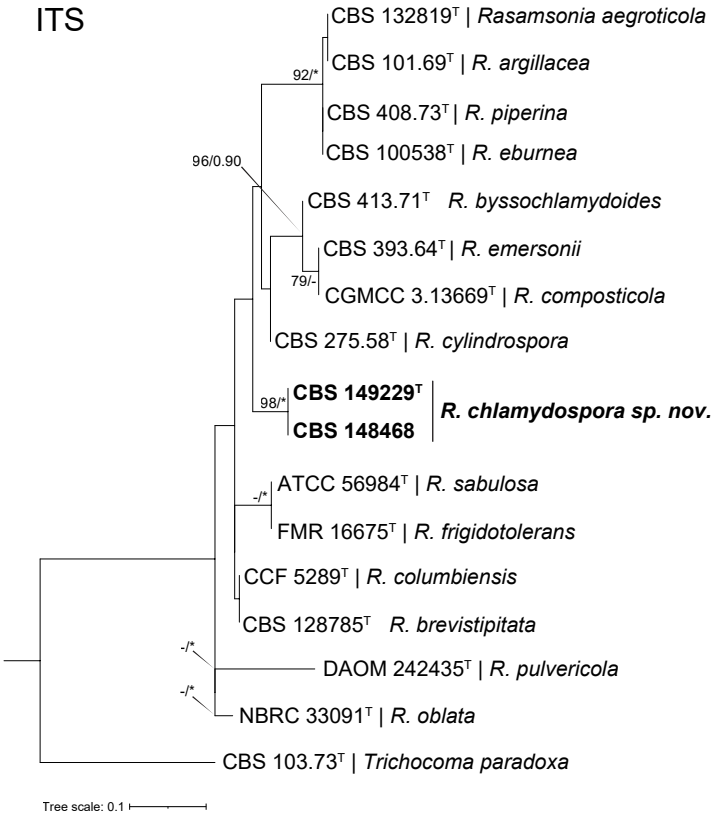

BenA

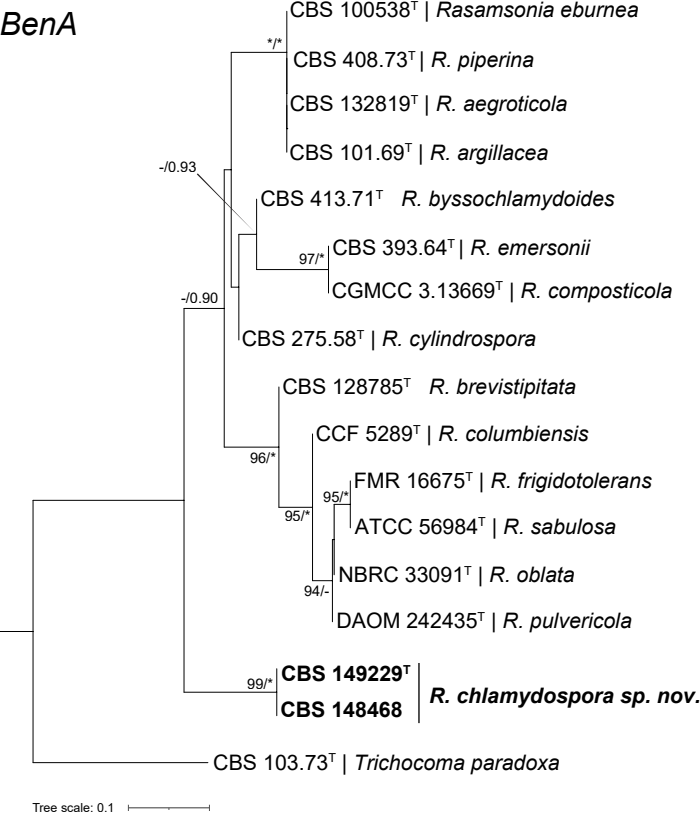

CaM

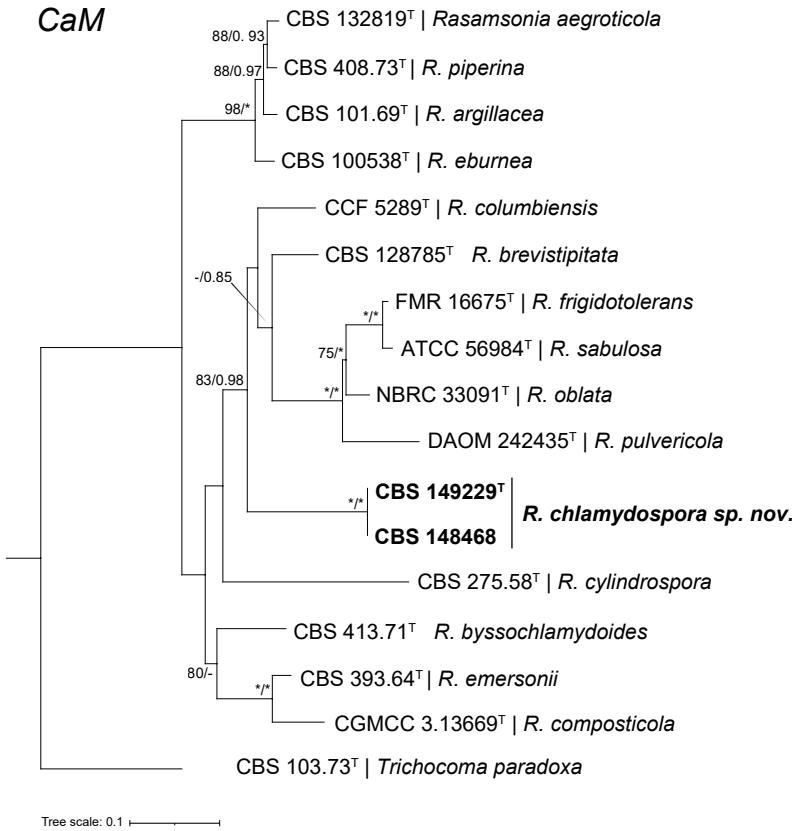

rpb2

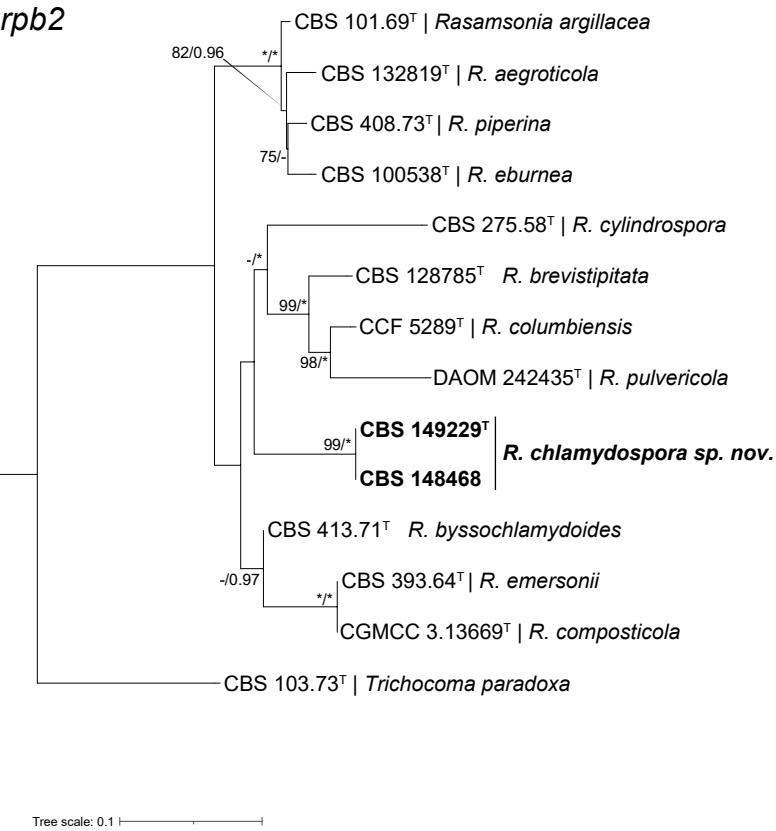

Supplement: Supplementary file 3 — Supplementary Figure S2. [file 41598_2023_42002_MOESM3_ESM.pdf]

# ITS

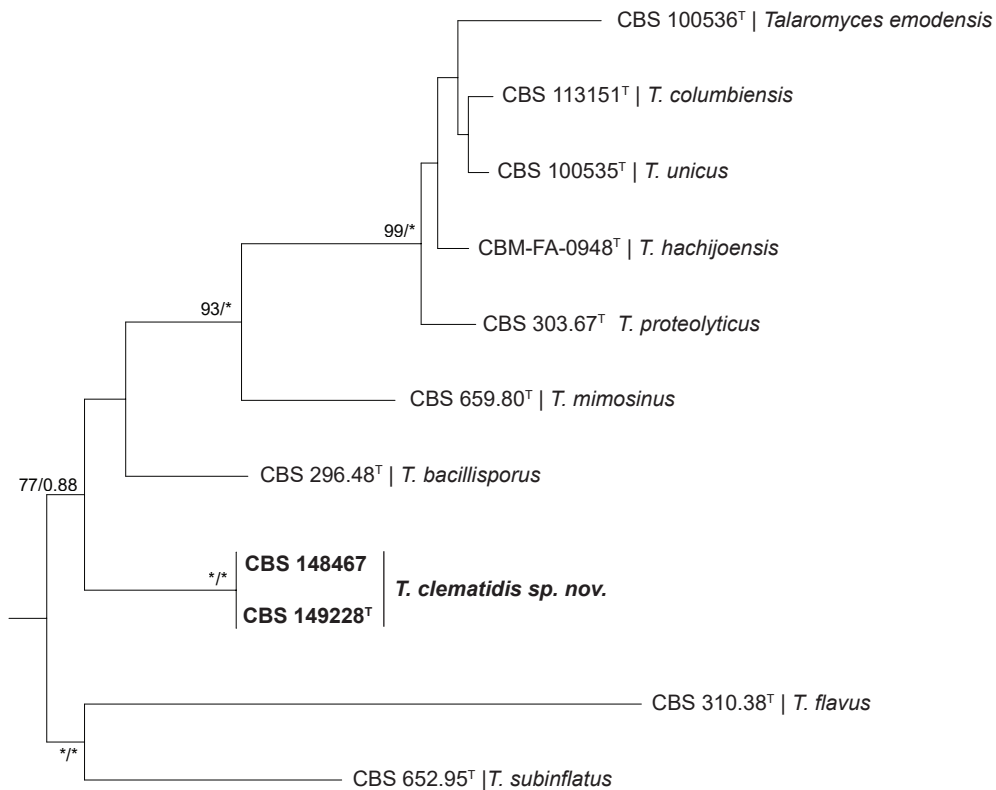

Tree scale: 0.1

Supplement: Supplementary file 4 — Supplementary Figure S3. [file 41598_2023_42002_MOESM4_ESM.pdf]

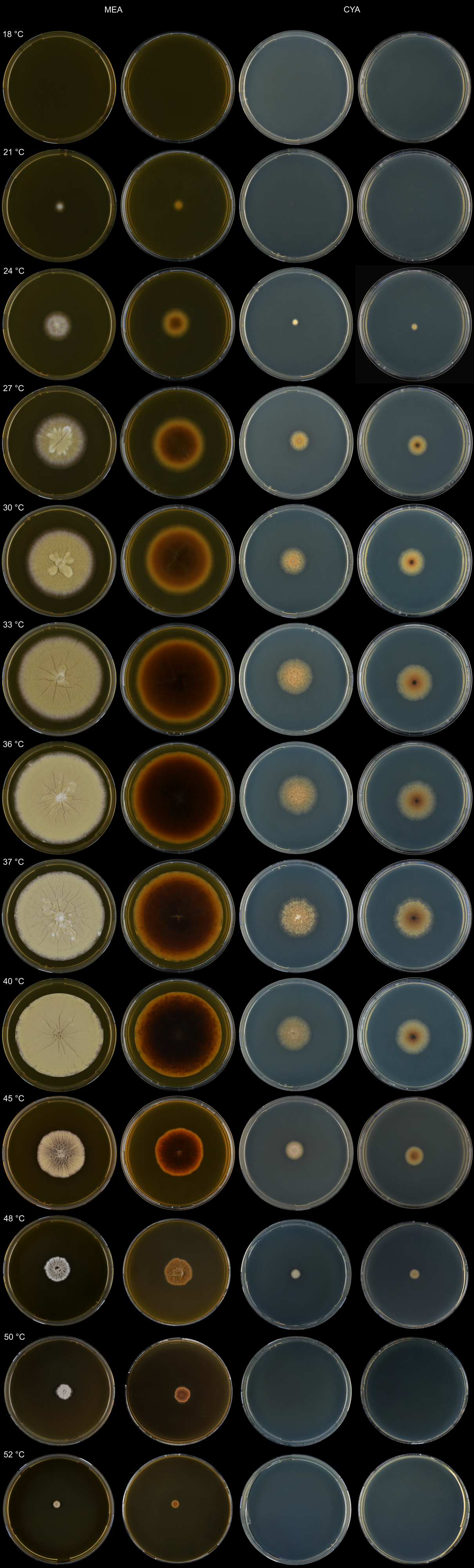

Supplement: Supplementary file 5 — Supplementary Figure S4. [file 41598_2023_42002_MOESM5_ESM.pdf]

7 days

14 days

ob.

rev.

ob.

rev.

DG18

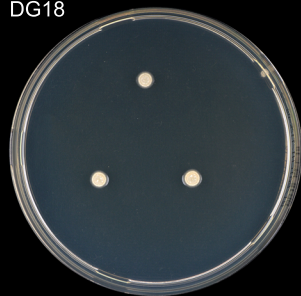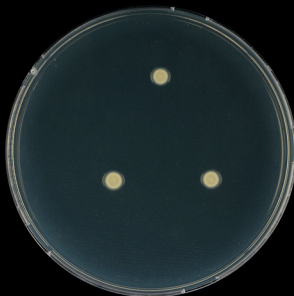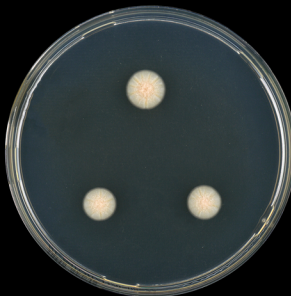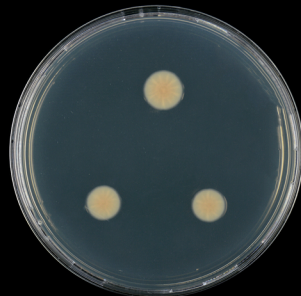

CYA

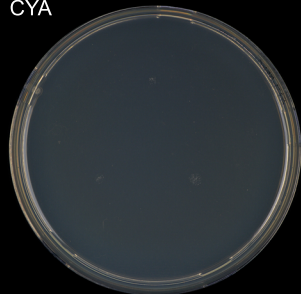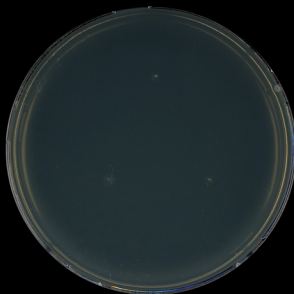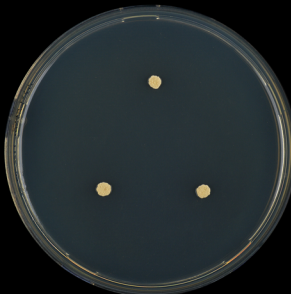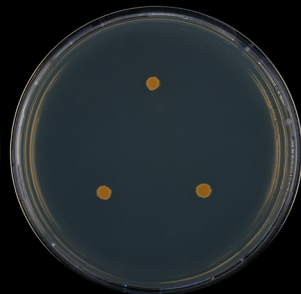

YES

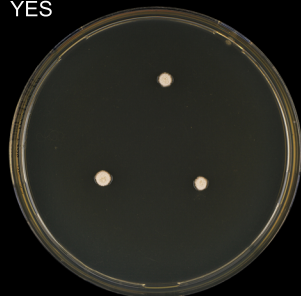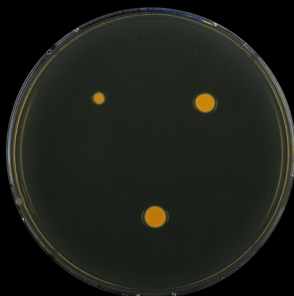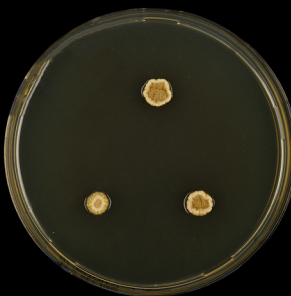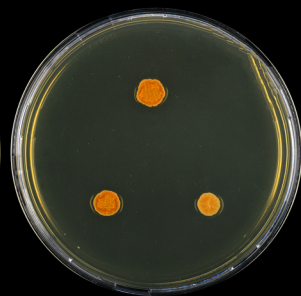

MEA

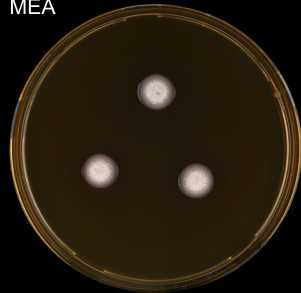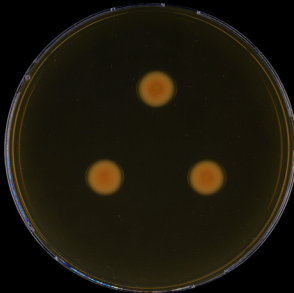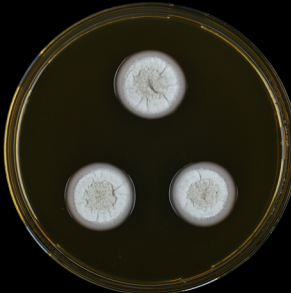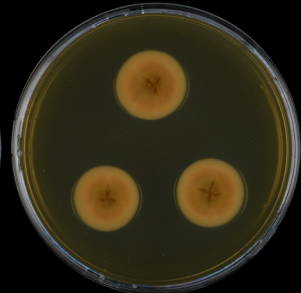

OA

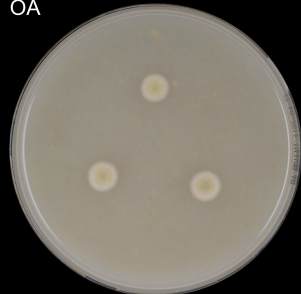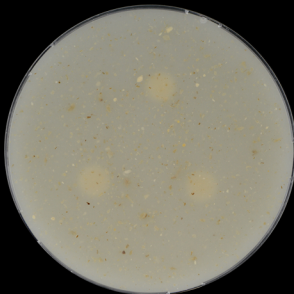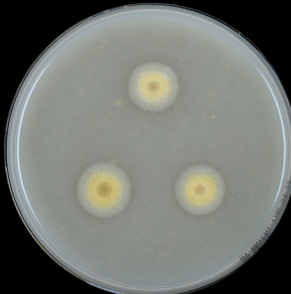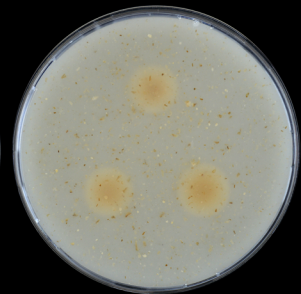

Supplement: Supplementary file 6 — Supplementary Figure S5. [file 41598_2023_42002_MOESM6_ESM.pdf]

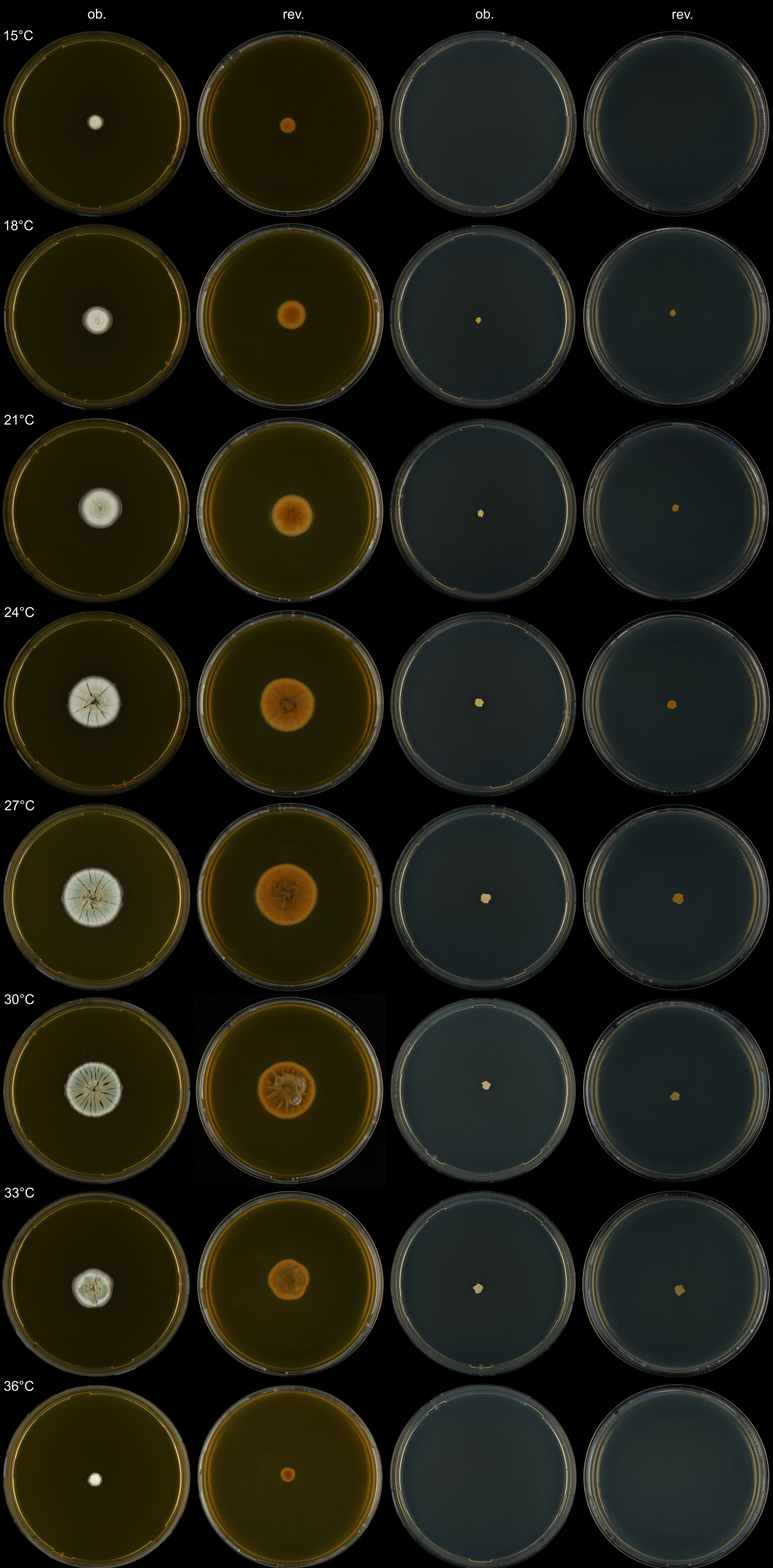

Supplement: Supplementary file 7 — Supplementary Figure S6. [file 41598_2023_42002_MOESM7_ESM.pdf]
